# Content validity, face validity and comprehensiveness of generic quality-of-life measures in adults and children with rare genetic conditions and their carers: a think aloud qualitative study

**DOI:** 10.1007/s11136-026-04324-7

**Published:** 2026-06-28

**Authors:** Mackenzie Bourke, Xuemin Zhu, Tiffany Boughtwood, Clara Gaff, Amy Hunter, Brendan Mulhern, Tessa Peasgood, Clare Stuart, James Buchanan, Ilias Goranitis

**Affiliations:** 1 Economics of Genomics and Precision Medicine Unit, Centre for Health Policy, Melbourne School of Population and Global Health, Melbourne, Australia; 2 Health Economics Research Centre, Nuffield Department of Population Health, University of Oxford, Oxford, UK; 3 Australian Genomics, Melbourne, Australia; 4 Murdoch Children’s Research Institute, Melbourne, Australia; 5 Melbourne Genomics Health Alliance, Melbourne, Australia; 6 Department of Paediatrics & Department of Medicine, Royal Melbourne Hospital, University of Melbourne, Melbourne, Australia; 7 Genetic Alliance UK, London, UK; 8 Centre for Health Economics Research and Evaluation, University of Technology Sydney, Sydney, Australia; 9 Division of Population Health, School of Medicine and Population Health, University of Sheffield, Sheffield, UK; 10 Mito Foundation, Sydney, Australia; 11 Health Economics and Policy Research Unit, Wolfson Institute of Population Health, Queen Mary University of London, London, UK; 12 National Institute for Health and Care Research Barts Biomedical Research Centre, London, UK

**Keywords:** Quality-of-life, Rare disease, Validity, Cognitive interview

## Abstract

**Purpose:**

This study aims to assess the content validity, face validity and comprehensiveness of the: (a) EQ-5D-5L, EQ-HWB, and ASCOT SCT4, for adults with rare genetic conditions; (b) the EQ-5D-5L, EQ-HWB, and ASCOT-carer for carers of adults or children with rare genetic conditions; and (c) the EQ-5D-Y-5L carer proxy-complete for children with rare genetic conditions.

**Methods:**

In total, 60 qualitative think-aloud interviews were conducted in Australia and England to understand individuals’ thought process during the completion of the QoL measures. Participants were subsequently led through a semi-structured discussion. Transcripts were analysed for whether participants demonstrated understanding of the measures and thematic analysis was conducted on responses to the semi-structured discussion.

**Results:**

The majority of participants showed good understanding and supported the validity of the measures for people experiencing rare conditions. For carers, however, a broader evaluative space than health-related QoL was preferred. Several non-health domains were identified as important to both patients and carers, including treatment availability, impact on employment and finance, information and uncertainty, medication and carer burden, impact of passing on a condition, relationships and social connection, and experience with the healthcare system.

**Conclusion:**

This study provides some support for the face validity and comprehensiveness of the measures for people experiencing rare conditions. However, several participants felt that the narrow health domains were inadequate to capture the breadth of their lived experience. Future research should explore the extent to which the measures capture differences and changes in the QoL domains identified as important to patients and carers.

**Supplementary Information:**

The online version contains supplementary material available at https://doi.org/10.1007/s11136-026-04324-7.

## Introduction

The measurement of quality of life (QoL) is integral to understanding the value of health technologies and guiding resource allocation. Economic evaluations of genomic technologies for rare genetic conditions have often focused on clinical outcomes, such as diagnostic yield, rather than QoL [1, 2]. This focus may reflect a perceived lack of relevance for QoL measurement or insufficient empirical evidence on the measurement properties of QoL instruments [3, 4]. The use of diagnosis related outcomes alone limits the scope of economic evaluation and inferences on cost-effectiveness.

Rare conditions are defined as those that affect fewer than 1 in 2,000 people [5]. They pose distinct challenges to the measurement and valuation of QoL There are over 7,000 rare conditions [5], which present in diverse ways [6], affecting various body systems to different extents [7, 8], and progressing at varying rates [8]. Approximately 80% of rare conditions are genetic in origin [9]. Due to their rarity individuals often face additional challenges, including limited information, support and access to services [10]. Rare conditions can significantly impact not only health, but also broader wellbeing domains of QoL [11, 12]. Furthermore, children and adults with rare genetic conditions often require considerable carer support which can have a substantial impact on carer QoL [13]. Carer impacts in economic evaluations have traditionally been captured as costs. However, there is growing support in the literature for capturing spillover effects of conditions on carer QoL, particularly in rare conditions where significant carer spillover effects have been demonstrated [12, 14]. To ensure adequate measurement of the impact of rare conditions and the potential value of interventions, it is necessary to understand whether QoL measures are valid and responsive for both individuals with rare conditions and their carers.

Due to the heterogeneity of genetic conditions, the scope for the development of a condition-specific QoL measure in this population is limited, and generic QoL measures are likely to be the most appropriate means to capture the diversity of experience [3]. However, evidence on the validity of generic measures for assessing health and broader QoL domains in this population remains limited. A recent scoping review identified 56 articles that measured QoL for 69 rare conditions [15]. While this suggests the potential validity of generic measures, empirical evidence on face and content validity is lacking [15]. As generic QoL measures attempt to capture impacts of a broad range of symptoms, there is evidence to suggest validity of carer-specific and generic QoL measures (namely the Carer Experience Scale, CarerQoL-7D, ASCOT-Carer, ICECAP-A, and EQ-5D-5L) for carers [16, 17]. However, existing evidence largely pertains to carers of adults with more common conditions, such as dementia, and may not be generalisable to rare conditions. To our knowledge, no studies have investigated the validity of these instruments for carers of adults or children with rare conditions.

This research aims to assess the content validity, face validity and comprehensiveness of 5 quality of life measures (EQ-5D-5L, the EuroQoL Health and Wellbeing Instrument (EQ-HWB – experimental long form), EQ-5D-Y-5L, and Adult Social Care Outcomes Toolkit (ASCOT-SCT4) and the ASCOT for Carers (ASCOT-C)), in adults rare conditions and carers of adults and children with rare conditions. The measures were selected to enable exploration, as each attempts to measure different constructs. Their specific focuses include health related QoL (EQ-5D-5L EQ-5D-Y-5L), carer quality of life (ASCOT) and broader wellbeing (EQ-HWB*)* [17–24]. Content validity and face validity were defined in line with the 2010 COSMIN international consensus [25] as the degree to which the content of the measure is an adequate reflection of the construct to be measured (Content validity) and the degree to which the domains of the measure look as though they adequately reflect the construct to be measured (Face validity) [25]. Comprehensiveness refers to whether no key domains are missing [26].

## Methods

Qualitative think-aloud interviews were used to understand the thought process of individuals as they completed the QoL measures [24, 27–29]. The measures completed by the participants are described in Table 1. Adults with rare conditions completed the EQ-5D-5L, EQ-HWB (including the EQ VAS) and ASCOT-SCT4 measures. Carers of children or adults experiencing rare conditions completed the EQ-5D-5L, EQ-HWB (including the EQ VAS) and ASCOT-Carer instruments. Carers of children also proxy-completed the EQ-5D-Y-5L and EQ VAS on behalf of their child. The EQ-5D-5L is one of the most widely used QoL measures in economic evaluation with demonstrated validity measuring health related QoL in diverse conditions [17–19]. The EQ-HWB™ is an experimental measure which considers broader wellbeing domains and has the potential to be relevant for individuals and carers. Initial evidence has suggested validity for capturing individual and carer QoL [20–22]. The ASCOT-SCT and SCT carer were designed for social care recipients, however have shown validity in capturing QoL among adult patients and carers, and has demonstrated convergent validity with the EQ-5D instruments [23, 24]. The EQ-5D-Y-5L was specifically designed for children and has demonstrated validity in several conditions, and high self- and proxy-complete correlation [30, 31].

**Table 1:** Quality-of-life measures and domains

Measure	Groups	Basis for inclusion	Domains
EQ-5D-5L^TM^ [44]	Adults Carers	Health related QoL One of the most widely used QoL measures in economic evaluation [19]. Demonstrated validity of measuring health related QoL in diverse conditions [18]. Has demonstrated validity for carer of adults with more common conditions [17]	Mobility Self-care Usual activities Pain/discomfort Anxiety/depression EQ VAS^a^
EQ-HWB (experimental version)^TM^ [45]	Adults Carers	This is an experimental quality of life measure which focuses on health, social care and carer related QoL The EQ-HWB has been demonstrated to be a valid instrument for capturing the difference in QoL between carers and the general public in China [20], and between parents of children with and without health conditions in Australia [22]. Domains including both health and broader wellbeing, as well as demonstrated validity in carers and adults makes it a reasonable candidate for inclusion [21, 22]	Vision Hearing Mobility Daily activities Self-care Sleep Exhaustion Loneliness Support Memory Concentration Anxiety Unsafe Frustration Sadness/depression Nothing to look forward to Control Ability to cope Acceptance Feeling good about self Ability to do things one wants Pain Level of pain Discomfort Level of discomfort
ASCOT-SCT4^TM^ [46]	Adults	Social care related QoL Has demonstrated validity in capturing QoL in adult patients with physical, sensory and mental health conditions [23] and convergent validity with the EQ-5D instruments [23]	Personal cleanliness and comfort Accommodation cleanliness and comfort Food and drink Safety Social participation and involvement Occupation Control over daily life Dignity
ASCOT-carer^TM^ [47]	Carers	Care-related QoL Has demonstrated validity in capturing QoL in caregivers of people with more common conditions (stroke, dementia, mental health conditions) [24]	Occupation Self-care Safety Social participation Control Encouragement and support Space and time
EQ-5D-Y-5L ^TM^ [48]	Carers (proxy completed by parents of children aged < 18 years, n = 26)	Child health related QoL (proxy complete) Has demonstrated convergent validity with the Peds-QL in patients who are acutely unwell, as well as those with beta thalassemia major, haemophilia and acute lymphoblastic leukemia [30]. has demonstrated high correlation between self-complete and proxy-complete in paediatric patients with beta thalassemia major, haemophilia and acute lymphoblastic leukemia [31]	Mobility Looking after myself Doing usual activities Having Pain or discomfort Feeling worried, sad or unhappy EQ VAS^a^

QoL, quality of life;

^a^Visual analogue scale—respondents are asked to rate subjective QoL from 0 (worst imaginable health) to 100 (best imaginable health)

### Sampling

Our study included two groups, adults with confirmed or suspected rare genetic conditions, and carers, who were parents of children with rare genetic conditions or adults with genetic intellectual disability. Participants were recruited from two countries, Australia and England, to enhance the generalisability of the findings. Australia and England were selected based on availability to authors of partner organisations which represent those with rare conditions. In Australia, participants who had consented to be recontacted after genomic testing under the Australian Genomics Rare Disease Flagships were emailed an invitation to complete an online screening survey. Respondents were provided with participant information and a consent form (Supplementary appendix 2). In England, Genetic Alliance UK distributed study information throughout their network, with interested participants being directed to the screening survey after contacting their national study leads (JB, XZ). After screening survey completion they were provided with the participant information and consent forms via email. An interview time was then organised. Purposive sampling ensured diversity in diagnostic status, condition, prognosis, treatment, phenotype, and patient reported QoL impact (using a 4-point scale). Minimum sampling quotas were also set for age and gender.

### Interviews

All interviews were conducted online using video conferencing software (either Zoom^TM^ or Microsoft Teams^TM^). The interviews in Australia were conducted between May and August 2023 by MB. Interviews in England were conducted between October and December 2023 by XZ, to allow recruitment gaps to be targeted with the English sample.

Each interview began with a brief introduction to the study, including reconfirmation of consent, followed by demographic questions and a practice think aloud question [32, 33]. Participants then completed the full QoL measures in random order whilst thinking aloud (Supplementary appendix 3).

Participant responses were monitored for the introduction of error. Error types were defined to remain consistent with previous literature [24, 34, 35]. These errors pertained to (1) General comprehension (i.e., whether the participant interpreted the question differently than the intended meaning); (2) Temporal comprehension (i.e., was the participant able to map the domain to the appropriate time period); (3) Decision response (i.e., did the participant give sufficient mental effort, as judged by the interviewer); and (4) Response process (i.e., did the participant understand the response scale and were they able to map their response to that scale) [24, 34, 35].

Following the completion of each of the measures a semi-structured discussion was led by the interviewer to further understand its content validity, face validity and comprehensiveness [25]. Participants were asked two semi-structured discussion questions, firstly, *“did this questionnaire reflect the way living with (caring for a child with) a rare condition impacts your quality-of-life?”*, and secondly, *“are there any other important considerations about the way your condition (caring) impacts your quality-of-life, that were not covered by this questionnaire?”* Participant responses to each question led to further discussion [36].

This research was designed collaboratively with Australian Genomics, the Australian Mito Foundation, Melbourne Genomics Health Alliance, and Genetic Alliance UK. This research project was approved by the Office of Research Ethics and Integrity at the University of Melbourne (2023-25881-39,273-5) and the Central University Research Ethics Committee at the University of Oxford (R78694/RE001).

### Analysis

To analyse the content validity, face validity and comprehensiveness of each of the measures thematic analysis was conducted on the interview transcripts using NVivo^TM^ software.

Thematic analysis is an accepted approach for understanding content and face validity in the COSMIN guidelines [36] and has demonstrated support in the literature [24, 37, 38]. The thematic analysis process was conducted separately on responses to each of the semi-structured discussion questions. Participants’ responses to whether they believed the instrument captured their QoL were recorded and tallied (Supplementary Fig. 1). Thematic analysis was conducted on the table using a bottom-up iterative approach [39] to maximise fidelity to the original data [40]. This approach utilised direct quotations from the transcript, which were coded by MB and XZ with closely matching descriptions of the quotes. These descriptions became the initial low-level codes. Initial codes were then iteratively grouped based on similarities into larger themes that accurately represented the data [39, 40]. Participant responses to whether there were any domains that were being excluded by the measures were tabulated (Supplementary Fig. 2). Thematic analysis was conducted on the tables using the same process as for the preceding question [39]. Themes were organised by individual measure. Quotations that adequately and concisely represented the themes were included in the results and tables.

The transcripts were analysed for the introduction of error into the response to the individual domains of the measures. Errors were classified based on analysis of the transcripts by two researchers (MB, XZ), with uncertainties resolved through discussion with senior researchers (IG, JB). Errors were tabulated by domain and instrument; the overall error rate was calculated as the number of errors per measure for the cohort. Upon the completion of their final measure participants were asked which was their preferred measure and why. The question was left open ended to allow participants space to justify their response. Responses were tallied and tabulated.

Analysis was conducted on the first 10 interviews then presented to the research team. Feedback was incorporated into the initial analysis and the remaining analyses were completed. Thematic analysis was conducted independently by two researchers (MB, XZ). To ensure rigour both researchers kept their thematic analysis blinded from the other until it was completed. At the completion of the analysis the wider research team discussed any differences, and themes were finalised through structured discussion.

Data from the Australian and UK samples were combined in the analysis. This reflected the aims of the study to understand the face and content validity of the instruments rather than comparing country specific perspectives. Given the comparatively smaller England sample which was purposively sampled to fill population gaps from the Australian sample, comparison was not appropriate, and pooling enhanced the depth of analysis.

## Results

### Participants

Overall, 60 interviews were completed, 30 with adults with rare genetic conditions and 30 with carers, 3 of which cared for adults with intellectual disability, 41 (68%) interviews were conducted in Australia and 19 (32%) in England. Table 2 shows respondents’ socio-demographic characteristics. The majority of participants had at least a bachelor degree (53%) and were female (78%). Participants reported 54 different primary diagnoses affecting a variety of body functions. The most commonly affected body systems reported included musculoskeletal, neurological, fatigue, cardiovascular, and digestive (Supplementary Figs. 3a and b). Most participants (68%) indicated that their disorder or caring responsibility had a large impact on their QoL, with a third (32%) of participants rating their prognosis, or the prognosis of the person for whom they care, as poor or very poor. All carers, including those of adults, were parents of the person for whom they care.

**Table 2:** Participant demographics (Australia and England)

Demographic table n (%)	Adults (n = 30)	Carers (n = 30)	Total Cohort (n = 60)
*Age*			
18–25	1 (3)	0	1 (2)
26–35	2 (7)	7 (23)	9 (15)
36–50	9 (30)	18 (60)	27 (45)
51–60	4 (13)	4 (13)	8 (13)
61–70	12 (40)	1 (3)	13 (22)
> 70	2 (7)	0	2 (3)
*Gender*			
Female	18 (60)	27 (90)	45 (75)
Education			
Bachelor or higher	14 (47)	18 (60)	32 (53)
*Diagnosis*			
Diagnosed	26 (87)	26 (87)	52 (87)
Suspected	4 (13)	4 (13)	8 (13)
*Impact*^a^			
No impact	1 (3)	0	1 (2)
Slight impact	2 (7)	3 (10)	5 (8)
Moderate impact	9 (30)	4 (13)	13 (22)
Large impact	18 (60)	23 77)	41 (68)
*Prognosis*^b^			
Very good	2 (7)	2 (7)	4 (7)
Good	9 (30)	6 (20)	15 (25)
Moderate	7 (23)	6 (20)	13 (22)
Poor	6 (20)	8 (27)	14 (23)
Very poor	2 (7)	3 (10)	5 (8)
I’m not sure	4 (13)	5 (17)	9 (15)

^a^The participants were asked “Which of the following best describes how your quality of life is affected by your genetic condition?”

^b^The participants were asked “How would you rate your prognosis?”

### Participant preferences

Participants in both groups indicated a preference towards the EQ-HWB, with 17 adults (57%) and 16 parents/carers (53%) choosing EQ-HWB as their most preferred measure (Supplementary Table 2). The ASCOT measures were the next most preferred in both groups, with 7 adults (23%) and 10 parents/carers (33%) preferring the respective ASCOT instrument.

### Content and face validity

As shown in Supplementary Table 3, for adult patients, most participants indicated that the measures could capture their quality of life (83% for EQ-HWB, 72% for ASCOT, 60% for EQ-5D-5L). For carers, the proportions were 40% for the EQ-5D-5L, 74% for the EQ-HWB and 97% for the ASCOT-carer. For the EQ-5D-Y-5L the proportion of parents who believed that instrument captured their child’s QoL was 76%.

Across both groups, participants who reported that an instrument captured their QoL gave similar reasons, noting that the instrument offered enough depth and relevance for them to accurately demonstrate the way their condition or caring responsibility impacts their life.

However, when participants reported that the instruments did not capture their QoL the reasons offered were more diverse (Table 4).

For adults with rare conditions several key themes emerged that were consistent across the instruments. Participants who believed the instruments did not capture their QoL reported there was a lack of depth for them to truly describe their experiences, stating that the instruments appeared *“very token”, “generic”* or *“not very granular”.* The second and third themes identified were a lack of condition-relevance, and a lack of rarity-relevance (Table 4). Some participants reported that instruments were not necessarily “*related to living with a genetic condition*” or could *“apply to somebody with a chronic disease that was not rare”.* The fourth theme that was identified was baseline adaptation. Some participants reported they had adapted their “*whole lifestyle… to [their] condition”* and were therefore able to complete their usual activities, however this did not capture the impact of the condition on their QoL. Finally, a fifth theme titled recall period too short was identified that was specific to the EQ-5D-5L and the EQ-HWB, with some participants reporting that they had “*good and bad times*” and that their conditions were therefore too dynamic to be captured within the short period of time specified by the measures (Table 3).

**Table 3:** Error rates

Measure	General comprehension	Temporal comprehension	Decision	Response	Total (%)
*Adults (AUS & ENG)*					
EQ-5D-5L (n = 30)	3	3	1	3	10 (6%)
EQ-HWB (n = 30)	6	0	0	1	7 (1%)
ASCOT SCT4 (n = 30)	3	0	0	5	8 (3%)
*Carers (AUS & ENG)*					
EQ-5D-5L (n = 30)	1	1	1	1	4 (2%)
EQ-HWB (n = 30)	4	2	0	2	8 (1%)
ASCOT-Carer (n = 30)	3	0	0	2	5 (2%)
EQ-5D-Y-5L (n = 26)	0	4	0	3	7 (5%)

For carers, a lack of question depth was the primary issue raised across all measures. Additionally, lack of caring-relevance, baseline adaptation, and recall period being too short were identified as reasons why the EQ-5D-5L and EQ-HWB were thought to not fully capture carer lived experience.

For carers of children, consistent themes emerged (Table 4). However, the challenge of proxy-completion in the presence of communication difficulties raised another challenge, as parents could not *“understand how they’re really thinking and processing”.*

**Table 4:** Did this questionnaire reflect the way living with (caring for a child with) a rare condition impacts your quality of life?

Adults	
All instruments	
Lack of depth	*“I mean, it [EQ-HWB] feels very token. Like it’s tick-a-boxy… I don’t think it can really explain the holistic nature of disability.”*—Adult, Australia *“The questions [ASCOT] are very generic and don’t really capture why people feel those things, what’s important to them.”—Adult, England* *“I think it’s [EQ-5D-5 l] not very granular, which might be too difficult I guess for people at the other end to sort of collect answers and for things to make clear sense. But I feel it’s just too vague, it doesn’t give people a true picture of really anything”*—Adult, Australia
Lack of condition-relevance	*“It’s a lifestyle questionnaire [EQ-HWB], but I don’t see how it’s related to living with a genetic condition. If I had a genetic condition or not, this would be the same questionnaire.”*—Adult, Australia *“It’s [ASCOT] not very specific… It wouldn’t give you a picture of the day-to-day struggles that a person has with their disability or condition. It’d give them a bit of general data but not a lot of detail.”*—Adult, Australia *“I can see that there are benefits in it [EQ-5D-5L]. But I wouldn’t have thought that that was necessarily relevant to me and my condition.”*—Adult, Australia
Lack of rarity-relevance	*“I don’t think it [EQ-HWB] was relevant to my rare condition. I would have to add that my rare condition, which is polycystic kidney disease is a particular genetic type.”*—Adult, Australia *“I would possibly, however say that it [ASCOT] could apply to somebody with a chronic disease that was not rare.”*—Adult, England *So I don’t think the questionnaire [EQ-5D-5L] is relevant to my rare condition. It is relevant to my quality of life, but not to my rare condition*.—Adult Australia
Baseline adaptation	“You know, if I was doing the activity of a normal person, then my answers would be probably different. So I would be in a lot more physical discomfort. Again, if I wasn’t on this treatment that I’m on, you know, again, my answers would be different. So it’s sort of—yeah, from a point of view, it’s my lifestyle change and my treatment plan are the ones that make it better than if it was without—if it was without any of the—if I didn’t have the treatment plan and if I did what a normal person would do, then yeah, it’d be a very different response.”—Adult, Australia [EQ-HWB] *“I think that again, that’s probably a big context for a lot of people with rare conditions that normal for me is not normal for someone else.”—Adult, Australia [ASCOT]* *“Well, I think it [EQ-5D-5L] just simplifies it a bit, because you know, when you have a rare—any rare condition, whether it’s genetic or it can be cancer or whatever it may be, you know, your whole lifestyle adapts to that condition, so that in some respects, it can be distorting, because for instance, if you have swollen joints, if you don’t do any activity, the pain is a lot less. So that’s usual. Because you make it usual”*—Adult, Australia
EQ-HWB & EQ-5D-5L	
Recall period too short	*“I think also just the last seven days as a thing, it’s kind of like—well, yeah, we have—I think with disability you can have—yeah, there are people that they’re often very static, but there’s often people that the majority of people with disability have good and bad days and they have good and bad times.”*—Adult, Australia [EQ-HWB] *“I don’t think it [EQ-HWB] would really differentiate that someone has a chronic condition. I think it would just be more about how that individual would be feeling over that seven days. And if I’m thinking about my long-term experience of having a chronic condition, this would probably reflect fluctuations in my quality of life over the experience of that condition.”*—Adult, Australia *“The problem with degenerative conditions is that they’re ongoing. It’s not like if you’ve lost a leg. You’ve lost your leg and it’s a one-off event. What I’ve got is an ongoing event and it’s changing all the time* *It’s also the case that what is normal for you is normal for you today, and that doesn’t tell you much about how it compares to someone of the same age and what you might call normal health”*—Adult, England [EQ-5D-5L]
Carers	
All instruments	
Lack of depth	*“Well because…, I suppose it’s [EQ-HWB] a little bit too generic. It’s not focused enough on the impacts that being a carer have. The things that I ticked or replied to probably would apply, even if I wasn’t caring for someone.”*—Carer, Australia *“I think some of the questions [in the ASCOT], they’re too general. So, like I said to you, they break down the examples but then it’s not letting you get across the variations in things, that parts may be okay, and parts might be not okay*.—Carer, England *“I feel like it’s [EQ-5D-5L] not really scraping the surface.”*—Carer, Australia
EQ-HWB & EQ-5D-5L	
Recall period too short	*“It’s [EQ-5D-5L] just one day—it’s a snapshot in time—it doesn’t consider more than today.”*—Carer, England *“I guess because it’s [EQ-HWB] been specific to the last seven days, like you don’t really know. That doesn’t really give you an overall idea because things could have been worse in the last seven days and they could have been better.”*—Carer, Australia
Lack of caring-relevance	*“So, if it [EQ-HWB] actually said, how does caring for your dependent influence your sleep patterns, that would be a different context to put around those answers.”*—Carer, Australia *“I think anybody could answer that [EQ-5D-5L] and, you know, it’s the kind of questionnaire that you might show up at your GP with if you were having ordinary anxiety or depression, for example. I don’t mean that in a rude way, but you could apply that to many different areas of life, rather than being the parent or carer of a person with a rare disease.”*—Carer, England
Baseline adaptation	*“Well usual activities is a vague question, because my usual activities have become caring and checking her twice daily, et cetera”*—Carer, Australia *“I guess there’s some words used in those that don’t make me immediately think of this because that’s like what I normally do on a normal day and that sort of thing.”*—Carer, Australia
EQ-5D-Y-5L	
Lack of child’s condition-relevance	*“I don’t see anything in that that would show the condition. You could ask that of any parent of any child, I don’t think it’d give you anything extra.”*—Carer, England *“No. Not unless you want to get more specific on incontinence and things like that”*—Carer, Australia
Recall period too short	*“The data it’s given me doesn’t reflect his daily life, because sometimes he’s a completely normal kid and other times he’s not.”*—Carer, England *“If they want to know generally how people are feeling, you have to get them thinking in a longer timeframe”*—Parent, Australia
Difficulty of proxy complete	*“It’s hard because she can’t communicate, I would say she’s happy. What are their underlying thoughts which is all a guess, really, because we can’t understand how they’re really thinking and processing.”*—Carer, Australia

### Error rate

Error rates (examples Supplementary Table 1) were low across all measures, ranging from 1 to 6% (Table 3) and there were no clear patterns to the errors that occurred. The highest error rate was for the pain/discomfort domain of the EQ-5D-5L, where there were 5 errors total (error rate 8%).

### Comprehensiveness

Table 5 shows the themes that emerged for adults with rare conditions regarding the domains not adequately captured by each of the instruments. In total, 17 domains were identified, of which seven were consistent across all instruments. The first theme was treatment availability. Participants reported that for some “*rare diseases there’s no treatment, and there’s no support”* and that has an impact on QoL that may not have been comprehensively captured by the domains of the instruments. The second theme was impact on professional opportunities, with some participants reporting that for those with rare conditions *“it was very hard to get work”* and this had an impact on QoL. Participants also noted that information/uncertainty about conditions may not have been adequately captured by the instruments, such as prognostic information about “*how quickly [their condition] might deteriorate”*, and that this was different for rare conditions compared to more common diseases. Participants felt that uncertainty impacted their QoL. Fourth, some participants reported that their conditions require onerous treatment regimens and for some participants the medication burden reportedly had *“the biggest impact”* on QoL, and some felt that this was not being fully captured. Fifth, participants felt that the impact of their experience of the healthcare system was significant considering they were constantly interfacing with *“professionals and healthcare bodies”* and that this was not captured by the instruments. Sixth, some participants reported they were most concerned about passing their conditions on to their children. Finally, some participants reported that the impact on *“relationships [was] not referred to”* by the instruments and this was a key driver of QoL.

**Table 5:** Domains not adequately captured by the measures

Adults	
Domain	Evidence
Availability of treatment	*“I know for a lot of disabilities there’s a lot of treatment and there’s a lot of support. And, again, in my particular case and in most other rare diseases there is no treatment and there’s no real support.”*—Adult, Australia [EQ-5D-5L] *“I think a questionnaire on that [treatment availability] would be really helpful… it’s a very long, hard road, and finding the right medical advice and support, allied health support is extremely difficult”*—Adult Australia [EQ-5D-5L] *“When it’s a rare disease, I mean, you know, from a PBS point of view, when you know, you’re one of what I believe is all of five people in the country that have this and those five people are my family, it’s sort of a case of you know, we’re never going to be on the radar at all”*—Adult Australia ASCOT
Impact on professional opportunities	*“The only thing is that it [ASCOT SCT4] didn’t say anything about work. I don’t know if that could be in this specific survey, but…”*—Adult, Australia *“It was very hard to get work. But if I had work, I would’ve had to change what I was doing probably because you get tired much quicker than you used to.”*—Adult, Australia [EQ-HWB] *“Maybe some employment stuff.”*—Adult Australia [EQ-HWB]
Information/uncertainty about condition	*“My condition is not like someone that has got cancer or a heart attack because everyone knows what happens with a heart attack or cancer. This one is not known, the disease that I’ve got, because no-one knows how quickly it might deteriorate or what other medical conditions I might face. That’s my concern.”*—Adult, Australia [EQ-HWB] *“There are things where a lot of people aren’t aware of mitochondrial disease and they don’t understand it; even healthcare professionals”*—Adult, Australia [EQ-5D-5L] *“I needed that information. A lot of people don’t want it, but I needed to have as much as I could, so that I knew if I wasn’t going to make it I could give my kids a place that they could go and find it.”*—Adult, Australia [EQ-HWB]
Medication/treatment burden	*“There’s not really any mention [EQ-HWB] of like, I don’t know if that’s relevant though. Medication, if that makes sense. I take quite a lot of medication every day. That’s probably the biggest impact on my life”*—Adult, Australia *“I think that, for my specific condition, my answers may have changed if there was one on there regarding medication side effects, but then not everyone takes medication so that could change a bit”*—Adult, Australia [EQ-5D-5L] *“I’m not sure it [ASCOT] quite sort of covers—what I’m sort of thinking about is how it feels to just have to allocate time for management. Like having to attend doctor’s appointments, then to schedule those for days off, having to work out, all of those sorts of things, just being in that kind of medical focus instead of just not worrying about that and just getting on with general life”*—Adult Australia
Worry about passing on condition	*“It’s something you’d think about all the time, about you know, the consequences of in my case, having kids, you know, will I or won’t I pass this on?”*—Adult, Australia [EQ-5D-5L] *“If we knew, would we have had kids, and I said what I know now, no. I would have adopted or done something else, I would not have had my own children.”*—Adult, Australia [EQ-HWB] *“So it’s a really hard situation to be in because, again, she [participant’s daughter] could be completely fine just carrying this condition or she could have a catastrophic event. So, there you have no control.”*—Adult Australia [EQ-HWB]
Relationships	*“Relationships can be very strained because of the situation that you are in. That’s maybe the only thing that I would suggest. I know, again, a lot of people don’t like talking about their relationship, but I’m in a fortunate position as I think I’ve said a lot of times where I’ve had great support from those around me. There may be a lot of people out there that their situation destroys those relationships. I don’t know. Just a thought”*—Adult, Australia [EQ-HWB] *“Relationships is not referred to [in the EQ-5D-5L]. Relationship with family members and relationship with medical support that you’re getting or not getting.”—*Adult, Australia *“Look, probably not. I think if I were in a different relationship, in a different house and I was a different person, and my illness progressed, then now things changed, I think”*—Adult Australia, ASCOT
Experience with healthcare system	*“I have also met parents with obviously with young children whose lives are consumed with healthcare. Every day is a healthcare day you know… if you were having to go to your GP on a fairly regular basis that still is—that obviously has an impact”*—Adult, England *“I think the quality of life when you’re living with a rare genetic disorder, very much depends on the quality of care that you receive from clinicians…”*—Adult, England *“I might be treated by different people fairly regularly. And so we have a conversation that starts again on a fairly regular basis…—a lot of people with rare diseases or any complex chronic conditions are constantly interfacing with, you know, professionals and healthcare bodies and I don’t think—I don’t think either of them have really dwelled a great deal on what my experience is of consuming healthcare on a regular basis”*—Adult, England
Parents/carers	
Carer burden	*“From my own experiences, probably always like a low hum of a burden in regards to care, and that can be, you know that might affect my physical or mental health. But generally it’s much more up and down, depending on the circumstances.”*—Carer, Australia [EQ-5D-5L] *“I think it’s the things that are taken for granted. Things like for me, I can’t just stop everything and go to the supermarket with my child. So because of his condition, I’m limited to what I can do, day to day. And he doesn’t have any—there’s no cure for what he has.”*—Carer, Australia [EQ-5D-5L] *“One of us has to be able to get enough sleep to be able to drive and transport the children somewhere so I can’t go on and take sleeping tablets you know to get my body into a routine of sleep, when there needs to be somebody to watch him on his feed overnight or his CPAP or whatever else, to make sure that he’s safe and, you know, doesn’t stop breathing overnight.”*—Carer, England
Financial impact	*“One thing that not one of them talked about is financial and the financial burden of caring for a child with a horrific illness is massive.”*—Carer, Australia [EQ-5D-5L] *“None of the questionnaires addressed the point of like financial cost of caring, so you know, has your caring caused your financial difficulty or have you had any big expenses in order to look after your child—none of the questionnaires addressed that. As in raising a disabled child often has hidden costs.”*—Carer, England [ASCOT] *“The impact on your income from having a child with special needs. It is massive. The lost income is, like, it’s just huge because, before I had [child], I was working full-time and I had always intended to return to work after some maternity leave, which I did. But, when I returned to work, I was not able to work anywhere near as many days as what I had intended to, and I also wasn’t able to access any daycare for him because his needs were too great.”*—Carer, Australia [ASCOT]
Condition severity	*“I suppose it probably should ask how [my child]’s condition impacts upon a carer. So today, yes, I’m feeling good today, I’m all that sort of stuff. Even if [my child] was sick today, I still feel fine today. So, it didn’t actually ask any questions in relation to me caring for [my child”]*—Carer, Australia, [EQ-5D-5L] *“If she was having a bit more of a down week, maybe some of my answers would’ve changed”*—Carer, Australia [EQ-HWB] *“Yeah. It certainly is certainly higher because of my child and his specific needs so that, you know, it’s just sort of on top of what most of us do just in our daily lives, for the people around us, our friends, our family, you know. Then you know the average. I suppose level. Then you put this whole extra chunk on top of that.”*—Carer, Australia [ASCOT]
Information/uncertainty about condition	*“I just think that it is really complicated, I suppose, dealing with this situation because it’s so rare and there’s not much information on it.”*—Carer, Australia [EQ-HWB] *“Because it’s [ASCOT], it’s not asking, because the biggest thing for me with our daughter is that I am often thinking about her future and how she’s going to go and the frustrations that it will affect what she wants to do with her life. There will be things that she can’t do”*—Carer, Australia *“Half the time it feels like they’re talking in French because you’ve got these exceptionally intelligent doctors who are using all of these words. So, I do a lot of research so I can try to speak the language and/or understand it.”*—Carer, Australia [ASCOT]
Support	*“No one can ever understand what it’s like. And I don’t think you can expect someone to fully understand what the day-to-day life is like, and I wouldn’t want my friends to have—I don’t want anyone to live with this but there’s something deep inside that you kind of just want to know someone that understands”*—Carer, Australia [ASCOT] *“I think just trying to gauge any limitations that we may be facing. Any sort of support that we have or don’t have, like on a day to day, like I guess just sort of to get an idea of what that looks like. Because I just feel like it’s not about, "Oh, you need a carers payment and you’ll be fine, you can just go about your day." There’s a lot that I feel like is neglected and I think maybe just trying to get a gauge on that would be a bit more beneficial.”*—Carer, Australia [EQ-5D-5L] *“Or just family support or—I know that you asked were there times you felt unsupported, but you could probably go a bit deeper into what actual supports do you have.”*—Carer, Australia [EQ-HWB]
Social engagement	*“I would say we didn’t cover any of the social elements of life which can be very isolating in families with a child with rare diseases. So yeah, understanding a bit of the isolation that can come into play.”*—Carer, Australia [EQ-5D-5L] *“Maybe [sighs] the social side of it. So if you feel isolated or, because I think finding your, well maybe it’s my current experience, but finding your kind of social group and where you fit, especially if you’re not working or um my children are primary school age so we’re out of the baby groups but I am technically still in a working age, so most of the people in my demographic are working. Yeah, so that social side of it, I think that’s quite an important wellbeing thing that’s not covered in there, whether you’re isolated or well-connected I think.”*—Carer, England *“The social, have you done anything for yourself in the last seven days? And has that impacted because you can’t, because you are caring for someone or? Yeah. I know you’ve got it, like, have you done anything that you’d like to do that you wanted to do? That sort of captures it, but maybe a little bit more detail.”*—Carer, Australia [EQ-HWB]
Availability of treatment	*“Well, he needs to take this medication but either medication isn’t very good, which one do you want to pick? I don’t have a medical background. You’re picking between a rock and a hard place, not knowing what the outcomes are. And I think that’s really hard too.”*—Carer, England *“I emailed a clinician who works at the University of Edinburgh because I’d like to have more information. I’ve emailed and I’m now on the radar for clinical and research opportunities at the Children’s Hospital of Philadelphia, because I just feel like there’s nothing for us, and I don’t want to be addressing [son]’s symptoms, and I don’t want us to kind of just be constantly hanging in there or constantly kind of just trying to be in this tunnel, and there’s light at the end of the tunnel but there’s just more tunnel.”*—Carer, Australia [EQ-5D-5L]
EQ-5D-Y-5L	
Community access	*“Probably as I said, like access to community. Yep. Access things. Maybe in terms of breaking it down maybe I say, because school and things like that I guess are in a different category to doing things like recreational activities. So school can be made a lot easier to do in terms of activities because if you go to say like an SSP [Schools for Specific Purposes] school, you do get travel and everything included in that, where if you’re doing more recreational things, you have that barrier of not having that extra help or extra access to go do things like that.”*—Carer, Australia
Frustration	*“Now, he hates feeling different. He hates not going to school. He hates that he has this and says, why do I have this? Why do I have to have this? Yeah. It’s like life isn’t fair.”*—Carer, Australia *“I think for my daughter as well, it can feel isolating having a rare disease. And I know what she finds really frustrating is people don’t understand why have you missed school for this appointment, or for what might appear a mild illness for other people. I think that can be really frustrating when people don’t understand.”*—Carer, England
Future issues	*“Yeah. It would be good to have a question about their future. Let me try and think about what I’m trying to actually say. Like, how they might feel about how it’s going to impact their life going forward. It doesn’t really address that”*—Carer, Australia
Social engagement	*“I think perhaps what’s missing is things like she doesn’t get included by her peer group with things like parties and sleepovers and playdates and she would love to be invited to more things like that and to be involved in more activities like that and she’s always asking for things like that but her children her age aren’t really interested in her because her mental age is about 50% lower than her actual age, so they’ve kind of outgrown her. So whilst they’re very protective of her and supportive, they don’t invite her to their houses to play or things like that and there’s a few of them that will come to our house but no, most of them really aren’t interested in playing with her.”*—Carer, England
Support	*“I sit down and we talk through it together, and by the end of a you know the conversation. He’s feeling better. So if you didn’t have someone that could give you that emotional support. Yeah, that little bit worried sad, unhappy could, could be. Could it be impacting you differently, I suppose… You know, to have a follow up question said, when I do feel worried, sad, or unhappy. I have someone I can talk to that, I trust, or whatever and kids I don’t know and this is sort of reflecting on kids in particular.”*—Carer, Australia
Treatment burden	*“You don’t know what their condition brings out in people or what it requires of them or is it when medication is administered, is it a difficult thing? Is it when appointments are—and procedures during appointments is that pain changes completely. And that’s not exactly day to day because you’re not going to have an appointment every day. But that doesn’t mean that the side effects or the residual shock and trauma from that appointment hasn’t carried on. So it’s a little bit hard to gauge it as like a general thing”*—Carer, Australia

Table 5 displays the themes that emerged for carers of adults and children with rare conditions regarding domains that may affect their QoL beyond the current domains of the measures. In total, 17 domains were identified, seven of which were consistent across all three instruments. Availability of treatment and information/uncertainty were consistent with those described by the adult cohort. Carer burden was a consistent concern: participants reported that the scale of the impact of their caring responsibility may not have been captured, as it was not specifically asked by the instruments. Financial impact was also consistently mentioned, with carers reporting that *“the financial burden of caring for a child with a horrific illness is massive”* and was a driver of their QoL. The fifth domain that was not adequately captured across all instruments was condition severity, which refers to the impact of the severity of the child’s condition on carers’ QoL. The sixth domain was support, which describes the impact of formal and informal support on carers’ QoL. The final domain was social engagement, which is the ability of carers to participate socially in their community. Participants described that caring can lead to social isolation that significantly impacts QoL.

The parents of children with rare genetic conditions identified 6 domains that were not adequately addressed by the EQ-5D-Y-5L. The first was community access, which describes the ability of the child to participate in the community. Carers described how “*doing things like recreational activities”* had a significant impact on the child’s QoL. Some participants highlighted that frustration was a significant factor in how their child feels that was not adequately captured by the instrument. A third domain was future issues, how a child feels about how their condition is *“going to impact their life going forward”* and what impact this has on the child’s current QoL. Social engagement was another domain that was not adequately captured, whether the child spends time with their peers in a recreational sense. Some carers reported that the impact of *“play”* with *“children [their] own age”* was a substantial consideration for their QoL. Support was considered by some carers to be a key driver of a child’s QoL but was not captured, that if a child *“didn’t have someone who could give”* that emotional support, this could negatively impact their QoL. The final domain was treatment burden, which refers to the burden of requiring frequent medical treatment or healthcare on the child’s QoL.

## Discussion

This is the first study to assess content validity, face validity and comprehensiveness of generic quality-of-life measures in people with diverse rare genetic conditions and their carers. Five instruments (EQ-5D-5L, EQ-HWB, EQ-5D-Y-5L, ASCOT SCT4, ASCOT-carer) were evaluated through think-aloud interviews in England and Australia with 30 adults and 30 carers of children and adults with rare genetic conditions.

For both adults with lived experience of rare disease and carers, most participants indicated that the measures could capture their quality of life, supporting the content and face validity of the instruments. This aligns with previous work showing rare diseases have a clinically and statistically significant impact on child and parental health-related quality of life [12]. However, twelve adults with conditions (40%) and 18 carers (60%) believed that EQ-5D-5L did not capture their lived experience. These responses highlighted the limits of current QoL measurement approaches in this population. The QoL measures considered were designed to be intentionally brief and cover important aspects of QoL that apply across conditions. It is therefore unsurprising that a portion of the participants felt that the measures lacked depth, condition-relevance, or rarity-relevance, and did not truly reflect how their conditions impacted their QoL [24, 32, 41]. Nevertheless, responses from both groups underscored the importance of non-health-related factors and their impact on QoL. This may explain why early work found limited evidence that existing instruments capture change in clinical, diagnostic and personal circumstances [4].

Given the significant heterogeneity in rare genetic conditions, developing a condition-specific instrument is impractical and there is naturally a trade-off between condition-relevance and generalisability of use [3, 6–8]. Furthermore, heterogeneity exists not only across rare conditions, but also within conditions. This research focused on relevance on measures across a broad set of conditions; however, it is plausible that performance of the measures would be better in some rare conditions than in others. It is therefore imperative to understand whether the domains included in generic QoL measures are adequate to differentiate between groups in this cohort with known differences in QoL, both within and across conditions, and the extent to which the measure can capture meaningful changes in what is important to patients and carers. Further research is needed to address this important gap.

Some participants reported that their condition, or caring responsibility had led them to make lifestyle changes which meant that questions that referred to what was *usual* for them, as asked in the EQ-5D-5L, and EQ-5D-Y-5L, may not accurately capture the impact on their QoL. This is difficult to interpret, QoL is subjective, and should reflect individual perceptions. It is reasonable for participants to believe that having a different baseline level of usual activities, which means that they can complete all their usual activities, does not adequately reflect the true impact of their condition on their QoL. However, it is also plausible that individuals who adapt their usual activities and can complete them experience higher QoL than if they continued being unable to complete previous activities. Similar issues have been noted in other QoL validity studies [42], and clinical evidence shows that changes in baseline usual activities can yield higher reported QoL through a process known as ‘*response shift’* [43]*.*

Comprehensiveness of the instruments was overall well supported. However, participants identified several additional non-health domains important to their QoL that were not directly captured. Treatment availability and information/uncertainty were identified by both individuals and carers. Adults with rare conditions were more concerned about the impact of their condition on employment, whereas carers were more concerned about the financial impact of caring. Adults reported that the impact of their condition on relationships was an important driver of QoL, while carers tended to place a greater emphasis on social connection. Adults reported that their concern about passing their condition on impacted their QoL, although carers—who were all parents—did not mention this as a concern. Finally, engagement with the healthcare system, whether positive or negative, had an impact on the QoL of adults with rare genetic conditions. Carers additionally identified carer burden as it relates to the severity of their child’s condition as an important aspect of their QoL that was not adequately captured by the instruments.

The domains outlined by participants highlight the breadth of the impacts of their conditions, or caring responsibilities on their QoL. Participants were asked broad QoL questions to capture all potentially relevant domains, however, the measures included in this study were not designed to capture QoL entirely, but rather to capture health-related QoL (EQ-5D-5L, EQ-5D-Y-5L), social care-related QoL (ASCOT SCT4), carer-related QoL (ASCOT) or a combination of health and wellbeing related QoL (EQ-HWB) [44–48]. These non-health domains therefore do not necessarily undermine the validity of the measures. Furthermore, variations in these domains may already be partially reflected in the existing domains, particularly those related to anxiety, stress, or social interaction, and may not require explicit inclusion. Future research should focus on establishing the construct validity and responsiveness of these measures for patients and families experiencing rare diseases.

## Strengths and limitations

This multi-country study used a flexible recruitment approach to achieve a diverse sample. Participants experienced a range of rare conditions, severities, and prognoses. This study assessed the content validity, face validity and comprehensiveness of new broader outcome instruments, and the think-aloud approach allowed for deep insight into the strengths and limitations of different instruments. Individuals with intellectual disability represent an important cohort of those with rare conditions, due to the nature of the think aloud interviewing no interviews were completed with adults with genetic intellectual disability, 3 interviews were conducted in carers of adults with intellectual disability. More inclusive interviewing practices have recently been developed, and future research could apply adaptive interviewing techniques to ensure this important cohort is not missed [49–51]. Our sample was on average highly educated and female, which may limit the generalisability. To limit participant burden only five instruments were used; important comparators may therefore have been excluded. While the approach applied in this research yielded valuable qualitative insights, formal conclusions about instrument validity, generalisability and responsiveness cannot be confirmed without additional quantitative analyses.

## Conclusion

Understanding QoL measurement in individuals with rare genetic conditions and their carers is essential for robust economic evaluation and accurate decision making. Establishing the validity and responsiveness of these generic QoL measures is therefore essential. The EQ-5D-Y-5L (proxy-complete), EQ-5D-5L, EQ-HWB and the ASCOT instruments showed signs of content and face validity for these groups with most participants reporting that the measures could capture their QoL. However, issues regarding how comprehensively several non-health domains were being captured were highlighted as important to individuals with conditions and carers. As comprehensive measurement of QoL was not the aim of these measures, these domains do not necessarily provide evidence against the validity of the measures, however further research is needed to assess whether the measures capture differences in these domains, and whether additional domains may be useful. While this study provided some support for the face validity of the measures, it also identified factors that may limit their ability to identify meaningful differences and changes in quality of life in these populations. Future research should focus on establishing the construct validity of these measures through known groups and convergence, as well as temporal effects, such as the responsiveness of the instruments to changes in diagnostic and clinical circumstances.

## Supplementary Information

Below is the link to the electronic supplementary material.

Supplementary Material 1

## Data Availability

The qualitative data supporting the findings of this study contain substantial identifying information and sensitive personal content from participants with rare conditions and their carers. To protect participant confidentiality, the full transcripts and raw qualitative data have not been made publicly available. De-identified excerpts relevant to the study’s findings may be made available upon reasonable request to the corresponding author, subject to institutional ethical approval and data sharing agreements.

## References

[1] Schwarze, K., Buchanan, J., et al. (2018). Are whole-exome and whole-genome sequencing approaches cost-effective? A systematic review of the literature. *Genetics in Medicine,**20*(10), 1122–1130. 29446766 10.1038/gim.2017.247

[2] Norris, S., Belcher, A., et al. (2022). Evaluating genetic and genomic tests for heritable conditions in Australia: Lessons learnt from health technology assessments. *Human Frontier Science Program,**13*(5), 503–522. 10.1007/s12687-021-00551-2 PMC9530105 34570356

[3] Lenderking, W. R., Anatchkova, M., et al. (2021). Measuring health-related quality of life in patients with rare disease. *Journal of Patient-Reported Outcomes,**5*(1), 61. 34283357 10.1186/s41687-021-00336-8 PMC8292508

[4] Pan, T., Wu, Y., et al. (2023). QALYs and rare diseases: Exploring the responsiveness of SF-6D, EQ-5D-5L and AQoL-8D following genomic testing for childhood and adult-onset rare genetic conditions in Australia. *Health and Quality of Life Outcomes,**21*(1), 132. 38087302 10.1186/s12955-023-02216-9 PMC10717517

[5] Murdoch Children’s Research Institute. Rare Genetic Disorders 2023 [Available from: https://www.mcri.edu.au/impact/a-z-child-adolescent-health/o-r/rare-genetic-disorders.

[6] Nguengang Wakap, S., Lambert, D. M., et al. (2020). Estimating cumulative point prevalence of rare diseases: Analysis of the Orphanet database. *European Journal of Human Genetics,**28*(2), 165–173. 31527858 10.1038/s41431-019-0508-0 PMC6974615

[7] Sequeira, A. R., Mentzakis, E., et al. (2021). The economic and health impact of rare diseases: A meta-analysis. *Health Policy and Technology,**10*(1), 32–44.

[8] Marwaha, S., Knowles, J. W., et al. (2022). A guide for the diagnosis of rare and undiagnosed disease: Beyond the exome. *Genome Medicine,**14*(1), 23. 35220969 10.1186/s13073-022-01026-w PMC8883622

[9] National Human Genome Research Institute. Rare Genetic Diseases 2018 [Available from: https://www.genome.gov/dna-day/15-ways/rare-genetic-diseases.

[10] Černe, T., Kragelj, L. Z., et al. (2024). Experiences of quality of life and access to health services among rare disease caregivers: A scoping review. *Orphanet Journal of Rare Diseases,**19*(1), 319. 39217366 10.1186/s13023-024-03327-2 PMC11365242

[11] Smits, R. M., Vissers, E., et al. (2022). Common needs in uncommon conditions: A qualitative study to explore the need for care in pediatric patients with rare diseases. *Orphanet Journal of Rare Diseases,**17*(1), 153. 35379257 10.1186/s13023-022-02305-w PMC8981675

[12] Wu, Y., Al-Janabi, H., et al. (2020). Parental health spillover effects of paediatric rare genetic conditions. *Quality of Life Research,**29*(9), 2445–2454. 32266555 10.1007/s11136-020-02497-3

[13] Sandilands, K., Williams, A., et al. (2022). Carer burden in rare inherited diseases: A literature review and conceptual model. *Orphanet Journal of Rare Diseases,**17*(1), 428. 36494728 10.1186/s13023-022-02561-w PMC9733280

[14] Al-Janabi, H., Efstathiou, N., et al. (2021). The scope of carer effects and their inclusion in decision-making: A UK-based Delphi study. *BMC Health Services Research,**21*(1), 752. 34325700 10.1186/s12913-021-06742-4 PMC8320027

[15] Delaye, J., Cacciatore, P., et al. (2022). Valuing the “burden” and impact of rare diseases: A scoping review. *Frontiers in Pharmacology*. 10.3389/fphar.2022.914338 35754469 PMC9213803

[16] Rand, S., Malley, J., et al. (2019). Measuring the outcomes of long-term care for unpaid carers: Comparing the ASCOT-Carer, Carer Experience Scale and EQ-5D-3 L. *Health and Quality of Life Outcomes,**17*(1), 184. 31842952 10.1186/s12955-019-1254-2 PMC6916016

[17] McLoughlin, C., Goranitis, I., et al. (2020). Validity and responsiveness of preference-based quality-of-life measures in informal carers: A comparison of 5 measures across 4 conditions. *Value Health,**23*(6), 782–790. 32540237 10.1016/j.jval.2020.01.015 PMC7532692

[18] Feng, Y.-S., Kohlmann, T., et al. (2021). Psychometric properties of the EQ-5D-5L: A systematic review of the literature. *Quality of Life Research,**30*(3), 647–673. 33284428 10.1007/s11136-020-02688-y PMC7952346

[19] Haraldstad, K., Wahl, A., et al. (2019). A systematic review of quality of life research in medicine and health sciences. *Quality of Life Research,**28*(10), 2641–2650. 31187410 10.1007/s11136-019-02214-9 PMC6761255

[20] Long, C., Mao, Z., et al. (2024). A head-to-head comparison of EQ-HWB and EQ-5D-5L in patients, carers, and general public in China. *Value in Health*. 10.1016/j.jval.2024.02.012 38447744

[21] Masutti, S., Falivena, C., et al. (2024). Content validity of the EQ-HWB and EQ-HWB-S in a sample of Italian patients, informal caregivers and members of the general public. *Journal of Patient-Reported Outcomes,**8*(1), 36. 38519577 10.1186/s41687-024-00706-y PMC10959916

[22] Bailey, C., Dalziel, K., et al. (2024). The validity of the EuroQol health and wellbeing short version (EQ-HWB-S) instrument in parents of children with and without health conditions. *PharmacoEconomics*. 10.1007/s40273-024-01351-5 38238605 PMC11168993

[23] Rand, S., Malley, J., et al. (2017). Validity and test-retest reliability of the self-completion adult social care outcomes toolkit (ASCOT-SCT4) with adults with long-term physical, sensory and mental health conditions in England. *Health and Quality of Life Outcomes,**15*(1), 163. 28821303 10.1186/s12955-017-0739-0 PMC5562982

[24] McLoughlin, C., Goranitis, I., et al. (2023). The feasibility and validity of preference-based quality of life measures with informal carers: A think-aloud study. *Value Health*. 10.1016/j.jval.2023.07.002 37516197

[25] Mokkink, L. B., Terwee, C. B., et al. (2010). The COSMIN study reached international consensus on taxonomy, terminology, and definitions of measurement properties for health-related patient-reported outcomes. *Journal of Clinical Epidemiology,**63*(7), 737–745. 20494804 10.1016/j.jclinepi.2010.02.006

[26] Mokkink, L. B., Herbelet, S., et al. (2025). Content validity: Judging the relevance, comprehensiveness, and comprehensibility of an outcome measurement instrument: A COSMIN perspective. *Journal of Clinical Epidemiology,**185*, Article 111879. 40562250 10.1016/j.jclinepi.2025.111879

[27] Janssen, L. M. M., Pokhilenko, I., et al. (2023). Methods for think-aloud interviews in health-related resource-use research: The PECUNIA RUM instrument. *Expert Review of Pharmacoeconomics & Outcomes Research,**23*(4), 383–389. 36880336 10.1080/14737167.2023.2187379

[28] Whitty, J. A., Walker, R., et al. (2014). A think aloud study comparing the validity and acceptability of discrete choice and best worst scaling methods. *PLoS ONE,**9*(4), Article e90635. 24759637 10.1371/journal.pone.0090635 PMC3997335

[29] Paul Mark, M., Fergus John, C., et al. (2020). Response process validity of three patient reported outcome measures for people requiring kidney care: A think-aloud study using the EQ-5D-5L, ICECAP-A and ICECAP-O. *British Medical Journal Open,**10*(5), Article e034569. 10.1136/bmjopen-2019-034569 PMC7232621 32414822

[30] Fitriana, T. S., Purba, F. D., et al. (2021). Comparing measurement properties of EQ-5D-Y-3L and EQ-5D-Y-5L in paediatric patients. *Health and Quality of Life Outcomes,**19*(1), Article 256. 34781978 10.1186/s12955-021-01889-4 PMC8591892

[31] Fitriana, T. S., Purba, F. D., et al. (2022). EQ-5D-Y-3L and EQ-5D-Y-5L proxy report: Psychometric performance and agreement with self-report. *Health and Quality of Life Outcomes,**20*(1), Article 88. 35659313 10.1186/s12955-022-01996-w PMC9164342

[32] Al-Janabi, H., Keeley, T., et al. (2013). Can capabilities be self-reported? A think aloud study. *Social Science & Medicine,**87*, 116–122. 23631786 10.1016/j.socscimed.2013.03.035 PMC3664929

[33] Willis GB. Cognitive interviewing: A tool for improving questionnaire design: sage publications; 2004.

[34] Murphy, M., Hollinghurst, S., et al. (2018). Qualitative assessment of the primary care outcomes questionnaire: A cognitive interview study. *BMC Health Services Research,**18*(1), Article 79. 29391003 10.1186/s12913-018-2867-6 PMC5796473

[35] Tourangeau R, Rips LJ, et al. The psychology of survey response. 2000.

[36] Terwee, C. B., Prinsen, C., et al. (2018). *COSMIN methodology for assessing the content validity of PROMs–user manual*. VU University Medical Center.

[37] van Leeuwen, K. M., Jansen, A. P. D., et al. (2015). Exploration of the content validity and feasibility of the EQ-5D-3L, ICECAP-O and ASCOT in older adults. *BMC Health Services Research,**15*(1), 201. 25976227 10.1186/s12913-015-0862-8 PMC4435604

[38] Engel, L., Kosowicz, L., et al. (2023). Face validity of four preference-weighted quality-of-life measures in residential aged care: A think-aloud study. *The Patient,**16*(6), 655–666. 37803217 10.1007/s40271-023-00647-6 PMC10570159

[39] Braun, V., & Clarke, V. (2006). Using thematic analysis in psychology. *Qualitative Research in Psychology,**3*(2), 77–101.

[40] Chapman, A. L., Hadfield, M., et al. (2015). Qualitative research in healthcare: An introduction to grounded theory using thematic analysis. *The Journal of the Royal College of Physicians of Edinburgh,**45*(3), 201–205. 26517098 10.4997/JRCPE.2015.305

[41] Engel, L., Bucholc, J., et al. (2020). A qualitative exploration of the content and face validity of preference-based measures within the context of dementia. *Health and Quality of Life Outcomes,**18*(1), 178. 32527264 10.1186/s12955-020-01425-w PMC7291594

[42] Dalziel, K., van Heusden, A., et al. (2023). A qualitative investigation to develop an adapted version of the EQ-5D-Y-3L for use in children aged 2–4 years. *Value in Health,**26*(10), 1525–1534. 37348834 10.1016/j.jval.2023.06.004

[43] Ortega-Gómez, E., Vicente-Galindo, P., et al. (2022). Detection of response shift in health-related quality of life studies: A systematic review. *Health and Quality of Life Outcomes,**20*(1), 20. 35123496 10.1186/s12955-022-01926-w PMC8818219

[44] Herdman, M., Gudex, C., et al. (2011). Development and preliminary testing of the new five-level version of EQ-5D (EQ-5D-5L). *Quality of Life Research,**20*(10), 1727–1736. 21479777 10.1007/s11136-011-9903-x PMC3220807

[45] Brazier, J., Peasgood, T., et al. (2022). The EQ-HWB: Overview of the development of a measure of health and wellbeing and key results. *Value in Health,**25*(4), 482–491. 35277337 10.1016/j.jval.2022.01.009

[46] Netten, A., Burge, P., et al. (2012). Outcomes of social care for adults: Developing a preference-weighted measure. *Health Technology Assessment,**16*(16), 1–166. 22459668 10.3310/hta16160

[47] Rand, S. E., Malley, J., et al. (2012). Adult social care outcomes toolkit for carers--German version. *PsycTESTS Dataset*. 10.1037/t85354-000

[48] Kreimeier, S., Åström, M., et al. (2019). EQ-5D-Y-5L: Developing a revised EQ-5D-Y with increased response categories. *Quality of Life Research,**28*(7), 1951–1961. 30739287 10.1007/s11136-019-02115-x PMC6571085

[49] Jones, P., Budd, L., et al. (2025). Towards a paramethodology: Exploring equitable methods for interviewing participants with alternative communication requirements about their experiences of air travel. *Journal of Transport & Health,**44*, Article 102150.

[50] McFarland, B., Bryant, L., et al. (2024). Adaptive interviewing for the inclusion of people with intellectual disability in qualitative research. *Journal of Applied Research in Intellectual Disabilities,**37*(1), Article e13182. 38044591 10.1111/jar.13182

[51] Luthra, R., Tideman, M., et al. (2025). ‘It may fit some; it may not fit others’: People with intellectual disability and their experiences of daily activity. *Scandinavian Journal of Disability Research*. 10.16993/sjdr.1173

