# Supplementary material for: Content validity, face validity and comprehensiveness of generic quality-of-life measures in adults and children with rare genetic conditions and their carers: a think aloud qualitative study
Source: Qual Life Res. 2026 Jun 28;35(8):221. doi: 10.1007/s11136-026-04324-7 (PMC13310824; doi:10.1007/s11136-026-04324-7)
Supplement: Supplementary file 1 — Supplementary Material 1 [file 11136_2026_4324_MOESM1_ESM.docx]

Supplementary Appendix 1: additional tables and figures

Supplementary Table 1: Error table

| Error type | Quote |
| --- | --- |
| General comprehension | *"I don't really get anxious. I get depressed. So, it's a bit hard to say both of them because anxiety doesn't come into it." – Adult, EQ-5D-5L*  *“Slightly difficult [to respond to vision question] because I do wear glasses” – Carer EQ-HWB* |
| Temporal comprehension | *“Well, maybe once or twice a month, so occasionally” – Carer EQ-HWB*  *‘Every now and then just a bit anxious about things” – Adult, EQ-5D-5L* |
| Decision | *“Unable to answer, too vague, how can you boil it down” [in response to VAS] – Adult, EQ-5D-5L*  *[Participant was unable to answer] " I don't know whether I've got to do blood tests and whether there's something going on" – carer, EQ-5D-5L* |
| Response | [Confusion as to the difference between Q8 and Q9] “I’d just guess” – Adult, ASCOT  “I do take issue with the last 2 questions” [unanswered] Adult- ASCOT |

Supplementary Table 2: Participant instrument preferences

| **Preference: n (%)** | **EQ-HWB** | **EQ-5D-5L** | **ASCOT/ ASCOT Carer** | **Not sure** |
| --- | --- | --- | --- | --- |
| **Adults** | 17 (57) | 2 (7) | 7 (23) | 4 (13) |
| **Parents/carers** | 16 (53) | 0 (0) | 10 (33) | 4 (13) |

Supplementary Table 3: Do you believe this instrument captures your lived experience (or the lived experience of your child)?

| **Capture n (%)** | **Yes** | **Yes/No** | **No** |
| --- | --- | --- | --- |
| **Adults** | | | |
| EQ-HWB | 19 (63) | 6 (20) | 5 (17) |
| EQ-5D-5L | 13 (43) | 5 (17) | 12 (40) |
| ASCOT* | 13 (45) | 8 (28) | 8 (28) |
| **Parents/carers** | | | |
| EQ-HWB | 17 (57) | 5 (17) | 8 (27) |
| EQ-5D-5L | 8 (27) | 4 (13) | 18 (60) |
| ASCOT-Carer | 23 (77) | 6 (20) | 1 (3) |
| EQ-5D-Y-5L^a^ | 10 (38) | 10 (38) | 6 (23) |

*1 participant missing due to error with recording equipment

a Proxy complete parents of children only (n=26)

Supplementary Table 4: Frequency of count data for body systems involved.

|  | AUS | | ENG | | Total | |
| --- | --- | --- | --- | --- | --- | --- |
| Number of body systems | Carers | Adults | Carers | Adults | Carers | Adults |
| 0 | 1 | 0 | 0 | 0 | 1 | 0 |
| 1 | 0 | 7 | 0 | 1 | 0 | 8 |
| 2 | 2 | 1 | 1 | 0 | 3 | 1 |
| 3 | 2 | 2 | 0 | 0 | 2 | 2 |
| 4 | 2 | 3 | 0 | 1 | 2 | 4 |
| 5 | 3 | 1 | 0 | 0 | 3 | 1 |
| 6 | 3 | 3 | 0 | 3 | 3 | 6 |
| 7 | 5 | 2 | 0 | 1 | 5 | 3 |
| 8 | 0 | 1 | 2 | 0 | 2 | 1 |
| 9 | 0 | 0 | 1 | 1 | 1 | 1 |
| 10 | 1 | 0 | 4 | 1 | 5 | 1 |
| 11 | 0 | 1 | 0 | 1 | 0 | 2 |
| 12 | 0 | 0 | 0 | 0 | 0 | 0 |
| 13 | 0 | 0 | 1 | 0 | 1 | 0 |
| 14 | 0 | 0 | 0 | 0 | 0 | 0 |
| 15 | 0 | 0 | 1 | 0 | 1 | 0 |
| 16 | 0 | 0 | 0 | 0 | 0 | 0 |
| 17 | 0 | 0 | 0 | 0 | 0 | 0 |
| 18 | 1 | 0 | 0 | 0 | 1 | 0 |


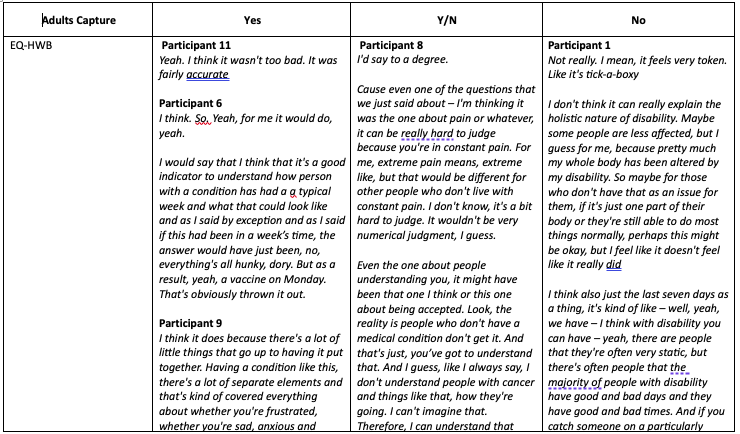
Supplementary Figure 1: Capture theme table


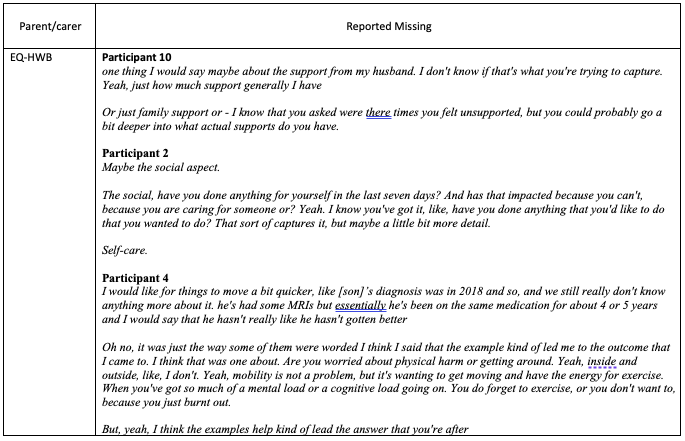
Supplementary Figure 2: Domains not adequately captured theme table

Supplementary Figure 3A) Frequency of body systems - adults

Supplementary Figure 3B): Frequency of body systems - carers

Supplementary Appendix 2: participant information and consent form

**Participant Information Statement**

| **HREC Project Number:** | HREC | | |  |  |
| --- | --- | --- | --- | --- | --- |
| **Short Name of Project:** | Psychometric evidence in rare diseases | | | |  |
| **Principal Researcher:** | A/Prof Ilias Goranitis | | | | |
| **Version Number:** | 1.0 | **Version Date:** |  | | |

Thank you for taking the time to read this **Participant Information Statement**. We would like to invite you to participate in a research project that is explained in this form.

This document is four pages long. Please make sure you have read all the pages.

**What is an Information Statement?**

An Information Statement tells you about the research project. It explains exactly what the research project will involve. This information is to help you decide whether you would like to take part in the research. Please read it carefully.

Before you decide if you want to take part or not, you can ask us any questions you have about the project.

**Taking part in the research is up to you**

It is your choice whether you take part in the research. You do not have to agree if you do not want to. If you decide you do not want to take part, it will not affect your relationship with The University of Melbourne, the University of Oxford, Australian Genomics, Melbourne Genomics, NHS GMS, or any other associated patient support networks

1. **What is the research project about?**

Health-related quality of life describes how a person perceives the impact of their physical and mental health on their day-to-day life. There are a number of tools currently available for measuring quality of life, however the effectiveness of these tools at encapsulating the lived experience of people with rare genetic conditions and carers of children with rare genetic conditions has not yet been established. This means that we are unsure whether real changes in the quality of life of these people would lead to changes in the values given by these measures.

This is important because government funding for medical tests and treatments depends, in part, on whether they lead to improvements in quality of life. Without an effective tool for measuring quality of life in people with rare genetic conditions, future funds they are entitled to may not be correctly allocated.

This project aims to assess how well 3 quality of life instruments (EQ-5D-5L, EQ-HWB and EQ-5D-Y-5L) accurately encapsulate the lived experience of adult patients and parents of children with lived experience of rare genetic conditions in Australia and England. It will involve asking participants to go through the questionnaires one at a time ‘thinking aloud’ as they go so, we can understand how they feel about the individual dimensions of each questionnaire. After this there will be a short discussion led by an interviewer asking participants how well they felt questionnaires captured their quality of life, an example of the type of question you may be asked is *“are there any aspects of living with the condition that impact on your life that you were not covered in the questionnaires?”.*

The results of this project will help us to understand how well currently available quality of life measures accurately encapsulate the lived experience of people with rare genetic conditions and carers of children with rare genetic conditions.

1. **Who is running the research project?**

The project is based at the University of Melbourne and the University of Oxford. A/Prof Ilias Goranitis is leading the study, working with James Buchanan, Tessa Peasgood, Brendan Mulhern, Tiffany Boughtwood, Clara Gaff, Clare Stuart, and Amy Hunter, with assistance from research assistants.

1. **Why am I being asked to take part?**

We are asking you to take part in the project because either

1. You have a lived experience of rare genetic condition or
2. You are the parent/caregiver of a child with a rare genetic condition
3. **What do I need to do in this project?**

Taking part in this project will involving attending an interview with a research assistant where you will be asked to fill out 3 or 4 questionnaires while thinking aloud. This will be followed by a 20-minute conversation about how well you believe the questionnaire captured your experience.

The sessions will be approximately 60 minutes in duration, held online via Zoom and will be audio and video recorded. If you do not want to be video recorded, you will be invited at the start of the session to turn your video off.

1. **Can I stop taking part in the project?**

You can drop out of the study at any point throughout this includes during he interview and immediately following provided it is before your data has been de-identified. Once your data has had your personal details removed it will be pooled with other data, at this point you will no longer be able to withdraw your data.

1. **What are the possible benefits for me and my family and other people in the future?**

There may not be any direct benefits to you or your child by taking part. However, this project will help us to know how best to measure quality of life for people with lived experience with rare genetic conditions. We hope that this will enable us to form stronger conclusions about the effectiveness of certain treatments and genomic testing. This will likely have an impact on future government spending and health service decision making and may lead to improved patient outcomes.

1. **What are the possible risks, side-effects, discomforts, and/or inconveniences?**

To take part in this research project, you will need to attend the interview online via zoom. This means that you will need access to a computer/tablet with internet, a microphone and a camera. This may be inconvenient for you, although you can choose the time that best suits you to attend. As a token of appreciation of your time we will provide you with a $50 voucher after the interview.

The questions asked on the questionnaires and in the subsequent discussion are generic, however they are related to your or your child’s health. This may be distressing for some participants.

1. **What will be done to make sure my information is confidential?**

In this project we will collect your responses but will not label them with your name or other identifying information so they will not be identifiable as yours. We will collect your name and contact information only for the purposes of organising and running your attendance at the interview. We will store your information securely at the University of Melbourne. We will store electronic information in secure databases. Once transcribed, the meeting notes and audio recording file will be deleted, however a copy of the recording will be stored on the secure University of Melbourne network drive.

The following people may access your information as part of this research project:

- The research project team
- The University of Melbourne Hospital Research Ethics Committee

We plan to publicise the findings of this research project in academic journals and at presentations such as conferences. These presentations could take place in Australia or overseas. Your information will be grouped together with that of the other participants taking part in the project. We will present the findings in such a way that you cannot be identified.

The University of Melbourne and the University of Oxford are research partners. This means that the two organisations will always share research information with each other.

1. **Will we be informed of the results when the research project is finished?**

At the end of the project, we will send you a summary of the project results if you request that we do so when you agree to take part.

1. **Who should I contact for more information or to take part in the research?**

If you have any questions about the research or would like more information you are also able to text, call or email:

**Name:** Mackenzie Bourke, Research Assistant, University of Melbourne

**Contact telephone:**  0419 399 132

**Email:** mackenzie.bourke@unimelb.edu.au

If you are willing to take part in the research, please make a time to attend one of the interview sessions by contacting Mackenzie Bourke by telephone or email.

| You can contact the Director of Research Ethics & Governance at The Royal Children’s Hospital Melbourne if you:   - have any concerns or complaints about the project - are worried about your rights as a research participant - would like to speak to someone independent of the project.   The Director can be contacted by telephone on (03) 9345 5044. The reference number for this project is 58713. |
| --- |

**Online Consent Form**

| **HREC Project Number:** | <insert HREC number here> | | |  |
| --- | --- | --- | --- | --- |
| **Short Name of Project:** | Psychometric evidence in rare genetic diseases | | | |
| **Version Number:** | 1.1 | **Version Date:** |  | |

- I have read this information statement and I understand its contents.
- I understand what I have to do to be involved in this project.
- I understand the risks I could face because of my involvement in this project.
- I voluntarily consent to take part in this research project.
- I have had an opportunity to ask questions about the project and I am satisfied with the answers I have received.
- I understand that this project has been approved by The Royal Children’s Hospital Melbourne Human Research Ethics Committee. I understand that the project and any updates will be carried out in line with the National Statement on Ethical Conduct in Human Research (2007).
- I understand I will receive a copy of this Information Statement.

| I do | I do not | Do you consent to taking part in this study? |
| --- | --- | --- |

| Participant Name |  | Date |
| --- | --- | --- |

Supplementary Appendix 3: Interview guide (Adult version)

Adults - Interview guide for Phase 1 qualitative interviews for face and content validity

1. **Welcome**

Interviewer introduces themselves

Interviewer asks questions unrelated to the research to put participant at ease and to help establish rapport

*How has your day been?*

*Where are you calling in from?*

*How’s the weather there?*

*Etc.*

**2.0 Introduction and checking consent**

Participants should already have received and signed consent form prior to the interview allowing participants ample time to consider the information provided and make an informed choice to consent.

Interviewer will check that participant has signed consent form prior to interview commencing.

*Before we start, I would like to check that you had a chance to look over the information sheet that we provided you via email.*

*Do you understand the purpose of this research?*

*Did you have any questions about anything?*

If participants have not read the PIS and signed the consent form interviewer will share screen and allow participants time to read the information and will sign on behalf of the participant.

*As you will have seen in the summary document researchers at the University of Melbourne and the University of Oxford are investigating how well currently available quality of life questionnaires capture the lived experience of people with rare genetic conditions. Right now, we’re in the initial phase of this work and we’re looking to find out the thought process of individuals as they complete these questionnaires.*

*You’ll be asked to complete 3 questionnaires. While you’re completing the questionnaires, we will also ask you to think aloud, so we gain some insight into how you are answering the questions. Before we begin the questionnaires, we will go through one practice example, and I’ll explain the process further.*

*After each questionnaire, we will have a short discussion where I will ask you questions about your thoughts on how appropriate you found the questionnaires.*

*It should take about 45-60 minutes in total.*

*Just so you know you are free to withdraw at any time – just let me know - and you do not have to offer a reason.*

*Finally, I will be using the record function through Zoom because it is difficult for me to take effective notes while conducting the interview. Some of the things you say during the interview may be used in anonymous quotes when we present our results in reports and publications.*

*Do you have any questions?*

*Are you happy to continue with the interview?*

**3.0 Sociodemographic Qs**

*The first questions I will ask are what we call demographic questions. They are about you and your life. The reason we ask is because we’re looking for a sample of people that most accurately represents a cross section of society and we want to make sure we don’t leave out any particular types of people.*

*These questions are by their nature very personal, so if you do not wish to answer them, please let me know or simply say “pass” and we will move on to the next question.*

*What is your current employment status?*

*Have you had any change to your employment status as a result of your genetic condition?*

*Are there any aspects other aspects of your life that you have had to change as a result of your genetic condition?*

*What is your highest level of education?*

*What is your postcode?*

**4.0 Practice Example**

*This is a think aloud interview and first we’re going to start with an example question that does not relate to quality of life.*

*I would like you to answer the question ‘How many windows are there in your home?’ While you answer the question, I would like you to talk me through your thought process.*

*For example, and this is just how my thoughts would go – you could very well approach it differently.*

*When I walk into my house I enter the lounge room, I look to my right, there are two large windows there, when I move into the lounge room, I can see the kitchen and there is one window above the sink so 3 etc.*

*Although there is likely a correct answer to this question, I’m not necessarily interested in that, I’m more interested in understanding how you came to reach you answer.*

*So, how many windows are there in your home?*

[interviewer may prompt participant to offer more information if necessary, during the practice question]

**5.0 Questionnaires and Interview**

*We’ll now begin going through the questionnaires.*

*In this part of the interview, like the practice example we are interested in the way you answer the questions and the process you go through to arrive at those answers. We’re trying to recreate the process you would go through if you were answering these questionnaires without me present, so if you believe a question is unclear, please tell me that, and answer it as best you can while explaining the process that you went through to reach that answer.*

*I will remain silent in the background except if there is a silence of more than a few seconds, in which case I will prompt you to ‘please continue to think-aloud.’ Thinking aloud is not a natural process for a lot of people so please don’t be surprised if you are prompted a number of times.*

*There is no rush to get through the questionnaires so please take as much time as you need to answer each question. After each questionnaire I’ll ask you how you felt about it. Please remember that for all of the questions you’re asked today there are no right or wrong answers we are simply interested in your thoughts.*

*We will now start the first questionnaire.*

*That is the end of the first questionnaire,*

1. *Did this questionnaire reflect the way living with a rare condition impacts your quality of life?*
   1. *Why?*
   2. *Why not?*
   3. *Follow up Qs based on response*
2. *Are there any other important considerations about the way your condition impacts on your quality of life, that were not covered by this questionnaire?*
   1. *Follow up Qs based on response*

*We will now start the second questionnaire.*

*That is the end of the second questionnaire*

1. *Did this questionnaire reflect the way living with a rare condition impacts your quality of life?*
   1. *Why?*
   2. *Why not?*
   3. *Follow up Qs based on response*
2. *Are there any other important considerations about the way your condition impacts on your quality of life, that were not covered by this questionnaire?*
   1. *Follow up Qs based on response*

*How is the interview going for you so far?*

This is an opportunity for positive feedback and to assess how the interviewer perceives the participant to be doing. It may be necessary to double check whether the participant wished to continue.

*We will now start the third and final questionnaire.*

*That is the end of the third questionnaire.*

1. *Did this questionnaire reflect the way living with a rare condition impacts your quality of life?*
   1. *Why?*
   2. *Why not?*
   3. *Follow up Qs based on response*
2. *Are there any other important considerations about the way your condition impacts on your quality of life, that were not covered by this questionnaire?*
   1. *Follow up Qs based on response*

*Well done, take a breath. That’s the hardest part of the interview done*

[provide relevant positive feedback]

*We have one final question; we would like you to consider all three questionnaires you completed today.*

*Out of those 3 questionnaires, which is your preferred one?*

- 1. *Why?*

**7.0 conclusion**

[Another chance to provide positive feedback]

*That brings us to the end of the interview.*

*Do you have any questions about anything that we went through?*

*Thank you very much for your time, the information you have provided is extremely valuable and will help us to understand quality of life better into the future. It may have important implication for future practice, policy and research.*

*I appreciate that you took time out of your day to speak with me and I’m extremely grateful.*

*Have a nice day,*

*Nice to meet you,*

*Etc.*
